# Substrate induced electronic phase transitions of CrI$_{3}$ based van der Waals heterostructures

**DOI:** 10.1038/s41598-020-80290-5

**Published:** 2021-01-08

**Authors:** Shamik Chakraborty, Abhilash Ravikumar

**Affiliations:** Nanoelectronics Research Laboratory, Department of Electronics and Communication Engineering, Amrita School of Engineering, Amrita Vishwa Vidyapeetham, Bengaluru, 560035 India

**Keywords:** Materials science, Nanoscience and technology, Physics

## Abstract

We perform first principle density functional theory calculations to predict the substrate induced electronic phase transitions of CrI$_{3}$ based 2-D heterostructures. We adsorb graphene and MoS$_{2}$ on novel 2-D ferromagnetic semiconductor—CrI$_{3}$ and investigate the electronic and magnetic properties of these heterostructures with and without spin orbit coupling (SOC). We find that when strained MoS$_{2}$ is adsorbed on CrI$_{3}$, the spin dependent band gap which is a characteristic of CrI$_{3}$, ceases to remain. The bandgap of the heterostructure reduces drastically ($\sim$ 70%) and the heterostructure shows an indirect, spin-independent bandgap of $\sim$ 0.5 eV. The heterostructure remains magnetic (with and without SOC) with the magnetic moment localized primarily on CrI$_{3}$. Adsorption of graphene on CrI$_{3}$ induces an electronic phase transition of the subsequent heterostructure to a ferromagnetic metal in both the spin configurations with magnetic moment localized on CrI$_{3}$. The SOC induced interaction opens a bandgap of $\sim$ 30 meV in the Dirac cone of graphene, which allows us to visualize Chern insulating states without reducing van der Waals gap.

## Introduction

Magnetism in two dimensions has been a fulcrum for many theoretical^1–3^, experimental and technological studies^4,5^ in the recent past. This is due to the degree of control offered by 2-D heterostructures enabling engineered levels of strain, surface chemistry, opto-electronic manipulation and detection of spin^6–10^. In this regard, the first two 2-D ferromagnetic crystals reported were Cr$_2$Ge$_2$Te$_6$^11^ and CrI$_{3}$^12^, discovered in 2017. Cr$_2$Ge$_2$Te$_6$ is a Heisenberg ferromagnet where the magnetic moments are oriented in all directions and has a very small magnetic anisotropy. CrI$_{3}$ on the other hand is an Ising A type antiferromagnet where the magnetic moments are oriented perpendicular to the 2-D plane^13^. With spin fluctuations significantly enhanced due to the crystal topology, these materials open new avenues to study low dimension magnetism. In the last few years alone, apart from these two materials, several other magnetic 2-D crystals have been discovered such as : FePS$_{3}$^14,15^, VSe$_2$^16^ and MnSe$_2$^17^. In this study we have investigated the substrate induced effects on the electronic properties of the resulting 2-D heterostructures by adsorbing graphene and MoS$_2$ on CrI$_{3}$.

Graphene^18^, a well studied two dimensional allotrope of carbon, is a non-magnetic semi-metal in its ground state and shows a negligible intrinsic spin-orbit coupling gap^19,20^. MoS$_2$^21,22^ on the other hand, shows significant influence to spin orbit coupling, a non-trivial semi-conducting bandgap and behaves as a topological insulator^23^. The study of the interfacial electronic and magnetic properties of graphene and MoS$_2$ adsorbed on CrI$_3$ offers the potential to design novel 2-D magnetic storage devices^24^. Typical magnetic storage devices include a ferromagnetic metal adsorbed on a heavy metal or a topological insulator^25^ with efficient spin-momentum locking and robust conversion of spin-current to charge current^26,27^. With spin fluctuations significantly enhanced due to the crystal topology, these 2-D heterostructures open new avenues to study low dimension magnetism^28–33^. In the case of CrI$_{3}$/MoTe$_{2}$, the heterostructure is modified to an intrinsic semiconducting ferromagnet with Curie temperature (T$_{c}$) of $\sim$ 60 K^34^. Further, the various emerging fields of technologies related to data storage^35^, energy generation^36^, water purification^37^ and biomedicine^38^, which were previously realized with bulk 3-D magnets, can now potentially be enhanced using 2-D magnetic heterostructures.

Apart from a host of fascinating fundamental properties that can be studied by these interfaces, there are various technological applications ranging from 2-D spintronics, magnonics and valleytronics^39–41^. The building block in the area of spintronics is a magnetic tunnel junction transistor where a very large tunneling magnetoresistance can be achieved by creating a sandwich of these heterostructures such as graphite-CrBr$_{3}$-graphite^42^. A spin field-effect transistor based on dual-gated graphene/CrI$_{3}$ tunnel junctions are shown to be less susceptible to interface imperfections, allow spin injection, control and detection^43^. The study of magnons and spinorbitronics involves exciting magnons from these magnetic interfaces and if the spin-orbit coupling is large, one can efficiently convert these travelling magnons into charge current voltage^44^. Besides design of novel vdw heterostructures, adsorbants like hydrogen (H) and oxygen (O) on CrI$_{3}$ is found to quench the magnetic moments of Cr atoms and introduce new dopant bands in the bandgap respectively^45^. Introducing periodic defects by adsorbing Lithium(Li) atoms on CrI$_{3}$ monolayer modifies the electronic properties of CrI$_{3}$ from a semiconductor to a half metal^46^. Multi-layered heterostructures such as monolayer WSe$_{2}$ and bi/trilayer CrI$_{3}$ showed layer-resolved magnetic proximity effects, where the field of proximity exchange is highly sensitive to the entire layered magnetic structure^47^. Despite these exceptional applications, there are several realistic challenges in magneto-electronic devices, particularly when designing spin-transfer torque magneto-resistive random-access memory. These include 2-D magnetism at room temperature, non-volatility and low power switching. Despite these challenges, 2-D heterostructure based magnetic memories are being researched extensively since 2017 as they offer better electronic control, perpendicular Ising anisotropy and efficient spin-torque magnetisation switching.

The motivation of this work is to understand the interfacial dynamics of CrI$_3$ based 2-D heterostructures formed by adsorption of MoS$_2$ and graphene. We perform *ab initio* Density Functional Theory (DFT) based calculations to determine the stable interfacial configurations and study the spin-dependent electronic and magnetic properties of the heterostructure. We further introduce spin orbit coupling in an attempt to further manipulate and engineer the electronic phase transitions. In the following section, we outline the computational methodology used. We then present our results and discuss them in the subsequent sections followed by the conclusion.

## Simulation methods

We perform *ab initio* Density Functional Theory (DFT) calculations within the generalized gradient approximation (GGA)^48^ framework using Perdew-Burke-Ernzerhof (PBE)^49^ exchange correlation functional and a plane wave basis set as implemented within the Quantum Espresso platform^50^. For spin polarized and spin orbit coupled (SOC) calculations, an ultrasoft scalar relativistic pseudopotential and an ultrasoft fully relativistic pseudopotential is used respectively. The kinetic energy cutoff of 50 *Ry* and charge density cutoff of 460 *Ry* are considered for both the heterostructures. A $12\times 12\times 1$ $\Gamma$ centered Monkhorst-Pack^51^ k-grid for a $1\times 1$ CrI$_3$ is used to sample the Brillouin zone and calculate the morphological and electronic structure relaxations. A denser $24\times 24\times 1$ $\Gamma$ centered k-grid for a $1\times 1$ CrI$_3$ is chosen to visualize the spin-dependent density of states (DOS) and bandstructure. The structural parameters are optimized until the Hellmann-Feynman force on all atoms is lesser than 10$^{-3}$ eV${}{\AA }^{-1}$. A large vacuum space of at least 18 ${}{\AA }$ is considered along the aperiodic z-axis for all the cases in order to avoid interaction between images^52^. The convergence criterion for the total energy is set to 10$^{-6}$ eV for spin-polarized calculations and 10$^{-5}$ eV for SOC calculations. Van der waals interactions are expected to play an important role in the system stability^52^ and Grimme correction^53^ is employed for this purpose. In order to identify the individual atomic contributions on the electronic spin dependent states, we also perform k-resolved DOS projected onto these states: $KDOS(k,E) = \sum _{\phi }\sum _n |\langle {\phi |\Psi _{nk}}\rangle |^2 \delta (E-\epsilon _{nk})$. Here $\phi$ are the wavefunctions centered around the individual atom types and used for Löwdin parametrization and runs over all the atoms of the heterostructure belonging to a particular atom type. $\Psi _{nk}$ and $\epsilon _{nk}$ are the Kohn–Sham wavefunctions and Eigen energies respectively of the heterostructure. Here the wavefunctions are summed over the *k* points of the Brillouin zone surface to extract information regarding the contribution of individual atom types to the electronic bands which can be validated by atom type projected DOS as well. To check the validity of the electronic bands obtained using GGA method, we have also performed calculations using GGA+U^54^ and HSE06^55^ hybrid functional methods. The effective on-site Coulomb interaction U$_{eff}$ = 3 eV and exchange interaction J = 0.9 eV^52^ were added according to Dudarev’s^56^ method for the Cr *d* orbitals within the GGA+U method.

## Results and discussions

**Figure 1:**
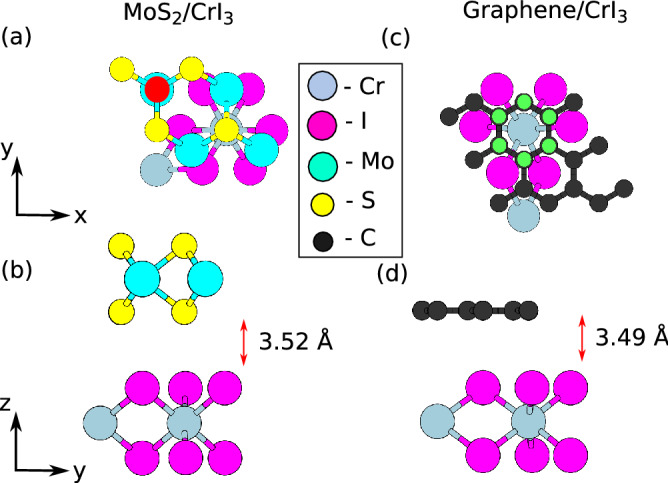
(**a**,**b**) The top and perspective view of the most stable configurations of 2:1 MoS$_2$ on CrI$_{3}$ respectively. The atom marked in red is the reference Mo atom with respect to which the various configurations have been named. (**c**,**d**) represent the same for 3:1 graphene on CrI$_{3}$. The graphene ring with respect to which we fix the nomenclature is highlighted by the green atoms.

We begin the study by investigating the most stable configurations of the two heterostructures by taking into account their translational and rotational symmetries. We calculate the adsorption energy $E_{Ads}$ of the system as $E_{Ads} = E_{l/CrI_3} - E_{CrI_3} - E_l$. Here $E_{l/CrI_3}$ is the total energy of the heterostructure after adsorption of graphene/MoS$_2$. $E_{CrI_3}$ and $E_l$ are the total energies of isolated CrI$_{3}$ and graphene/MoS$_{2}$ respectively. The 2:1 MS/CrI$_{3}$ system comprises of a $2\times 2$ MoS$_{2}$ adsorbed on a $1\times 1$ CrI$_{3}$. Based on the crystalline symmetry, we find three probable stable morphologies for 2:1 MS/CrI$_{3}$ case: Top, Hollow and Bridge configurations as summarized in Fig. 1 of supplementary information (SI)^57^. The supercell geometries are defined based on the position of the top-left Mo atom with respect to the CrI$_{3}$ layer below (as visualized using Xcrysden^58^ and shown in Fig. 1a). The most stable configuration is when top-left Mo atom is at the center of the ring created by the I atoms of CrI$_{3}$ (Hollow configuration) as shown in Fig. 1a,b. The adsorption energy value was found to be − 517 meV^34^ and the interlayer distance is 3.52 ${}{\AA }$. The top and hollow configurations have very similar adsorption energies and interlayer distances. We therefore performed an additional relaxation calculation using fully non-local exchange correlation functional using localized basis set within SIESTA^59^ to confirm that the hollow configuration was the most stable one (by 0.002 eV). The adsorption energy values for the Top, Bridge and Hollow configurations are summarized in Supplementary Table 1 of SI^57^. The relaxed lattice constant of the supercell is 6.68 ${}{\AA }$ with a strain of 7% distributed on both the layers. The equilibrium interlayer distance is found to be 3.52 ${}{\AA }$. The 3:1 G/CrI$_{3}$ is a $3\times 3$ unit cell of graphene adsorbed on $1\times 1$ CrI$_{3}$. We identify four possible configurations based on the crystalline symmetry of the layers: Top, Hollow, Bridge and Top-y. The nomenclature used is with respect to the position of the central graphene ring (shown by the green atoms of Fig. 1c) and CrI$_{3}$ lattice below. The top and the hollow configurations were found to be identical and the adsorption energies and relevant geometric parameters have been summarized in the SI^57^. The top configuration where the highlighted graphene ring shown in Fig. 1c is placed on top of Cr atom is found to be most stable with an adsorption energy value of − 677 meV^52^. The relaxed lattice constant is 7.194 ${}{\AA }$ and the interlayer distance is 3.49 ${}{\AA }$. The supercell accounts for a uniform strain^60^ of 2.5% distributed on both layers.

### Electronic structure of 2:1 MS/CrI$_{3}$

We now discuss the electronic and magnetic properties of 2:1 MS/CrI$_{3}$ by calculating the spin dependent density of states (DOS) and bandstructure. We further introduce spin-orbit coupling to understand its influence on the electronic structure of the chosen heterostructure.

**Figure 2:**
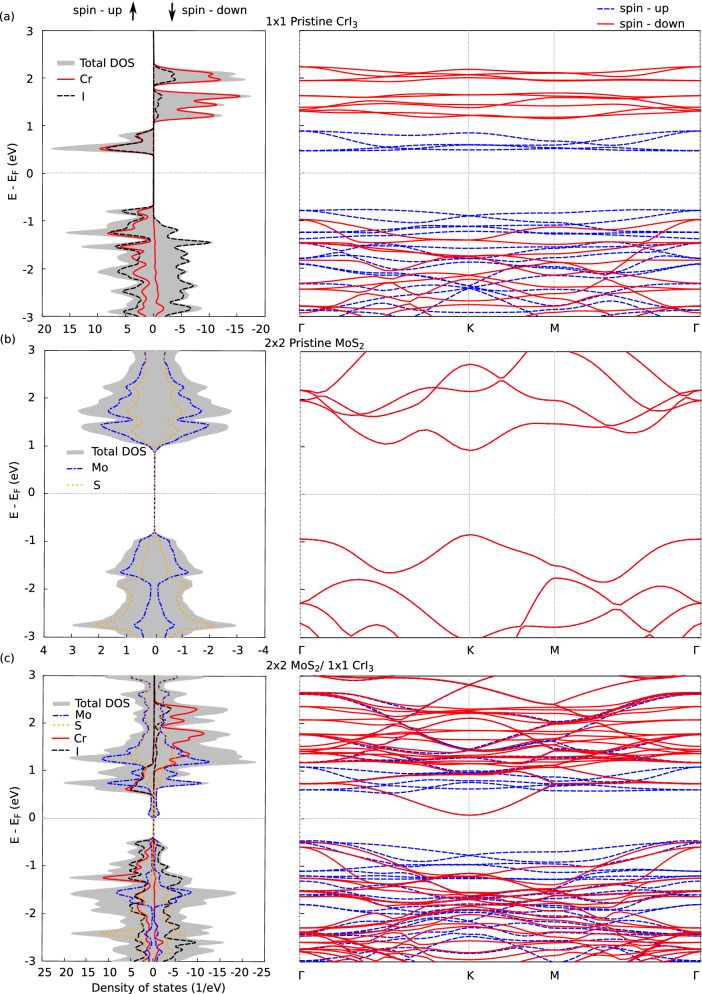
Spin-polarized projected density of states (DOS) and bandstructure for (**a**) pristine monolayer of $1\times 1$ CrI$_{3}$ (**b**) pristine (unstrained) monolayer of $2\times 2$ MoS$_{2}$ (**c**) 2:1 MS/CrI$_{3}$ (Hollow configuration) with $2\times 2$ MoS$_{2}$ adsorbed on $1\times 1$ CrI$_{3}$. Gaussian smearing of 0.004 eV has been used to visualize the DOS.

Figure 2a (Left panel) shows the spin-dependent DOS of pristine monolayer $1\times 1$ CrI$_3$ and the system wavefunctions projected on individual atom types (Cr and I). A $\Gamma$ centered k-grid of $24\times 24\times 1$ and a Gaussian smearing of 0.004 eV has been used to visualize the DOS. The right panel of Fig. 2a shows the spin-dependent bandstructure of monolayer CrI$_{3}$ along the k-path $\Gamma$-K-M-$\Gamma$ spanning the first irreducible Brillouin zone. CrI$_{3}$ is an indirect bandgap ferromagnetic semiconductor^61^ with a spin up bandgap of 1.24 eV and 2.11 eV for the spin-down states which is in agreement with other theoretical^61^ and experimental studies^62^. The bandgaps calculated using GGA+U method for the spin up and spin down states are 1.02 eV and 3.3 eV respectively which are in close agreement with reported values^34,63^. The bandgap value calculated using HSE06 is 2.12 eV^52^ and these values are compared and tabulated in Table 2 of SI^57^. The magnetic moment of Cr is $\sim$ 3 $\upmu _{\mathrm{B}}$/atom and that of I is $\sim$ 0.06 $\upmu _{\mathrm{B}}$/atom, which is consistent with the saturation magnetization of bulk CrI$_{3}$^64^. It is worth noting that spin-dependent nature of the bandgap for pristine CrI$_{3}$ with the spin-up states close to the Fermi level have equal contributions from both Cr and I.

The spin dependent DOS and bandstructure for unstrained $2\times 2$ MoS$_{2}$ is shown in Fig. 2b. Pristine MoS$_2$ is non-magnetic with a direct bandgap of 1.77 eV which is in good agreement with experimental^65^ and theoretical^66^ studies. Adsorption of MoS$_2$ on CrI$_{3}$ induces a strain of 7% on both the layers and has been uniformly distributed when constructing the supercell. The bandgap of monolayer strained MoS$_2$ is found to exhibit a direct to indirect bandgap transition and the quantitative value of the bandgap is found to reduce with increasing values of uniaxial strain^67^. This is due to the phonon softening with increased strain which breaks the degeneracy of the E’ Raman modes of strained monolayer MoS$_{2}$^67^. Due to the weak van der Waals interaction at the interface of CrI$_3$ and MoS$_2$, we observe a similar transition of MoS$_2$ bands as seen in Fig. 2c.

In Fig. 2c, we have shown the total spin-dependent DOS, its projections on individual atom types (left panel) and bandstructure of 2:1 MS/CrI$_{3}$ system for both its spin states (right panel). It can be observed from the DOS that the heterostructure remains magnetic with the magnetic moment largely localized on CrI$_{3}$. The magnetic moment on CrI$_{3}$ remains relatively unchanged (with respect to its pristine counterpart) with $\sim$ 3 $\upmu _{\mathrm{B}}$/atom on Cr and $\sim$ 0.06 $\upmu _{\mathrm{B}}$/atom on I. This points to a weak van der Waals interaction between the interface inhibiting interlayer magnetic exchange. Mo and S have very negligible magnetization displaying atomic magnetic moments of $\sim$ 0.0005 $\upmu _{\mathrm{B}}$/atom and $\sim$ 0.0004 $\upmu _{\mathrm{B}}$/atom respectively. From the DOS shown in Fig. 2c, one can observe that the bandgap of the system reduces significantly (when compared to their pristine counterparts) to 0.53 eV for both the spin-up ad spin-down states. Above the Fermi level, the states of Mo primarily shift closer to the Fermi level and the states of Cr and I remain at relatively unchanged energies (between 0 and 1 eV). One can also observe a broadening of these states suggesting a weak interlayer hybridization. Below the Fermi level, we observe a relatively strong mixing of atomic states and a collective shift of these states closer to the Fermi level.

The electronic transition of a reduced indirect bandgap of 0.53 eV for both the spin-up and spin-down states as seen in the bandstructure of Fig. 2c can be attributed to two primary reasons: The presence of a strained MoS$_{2}$ and the weak interlayer interaction with CrI$_3$. The bandgap values calculated using GGA+U are found to be similar with GGA approximations as tabulated in Table 2 of SI^57^. This can be further confirmed by performing Löwdin population analysis^68^ to quantify the interfacial charge transfer. It is found that MoS$_{2}$ loses a negligible atomic charge of 0.051 electrons and CrI$_3$ gains a similar value of electronic charge of 0.058 electrons. The detailed orbital projected DOS for 2:1 MS/CrI$_{3}$ has been included in the SI^57^.

**Figure 3:**
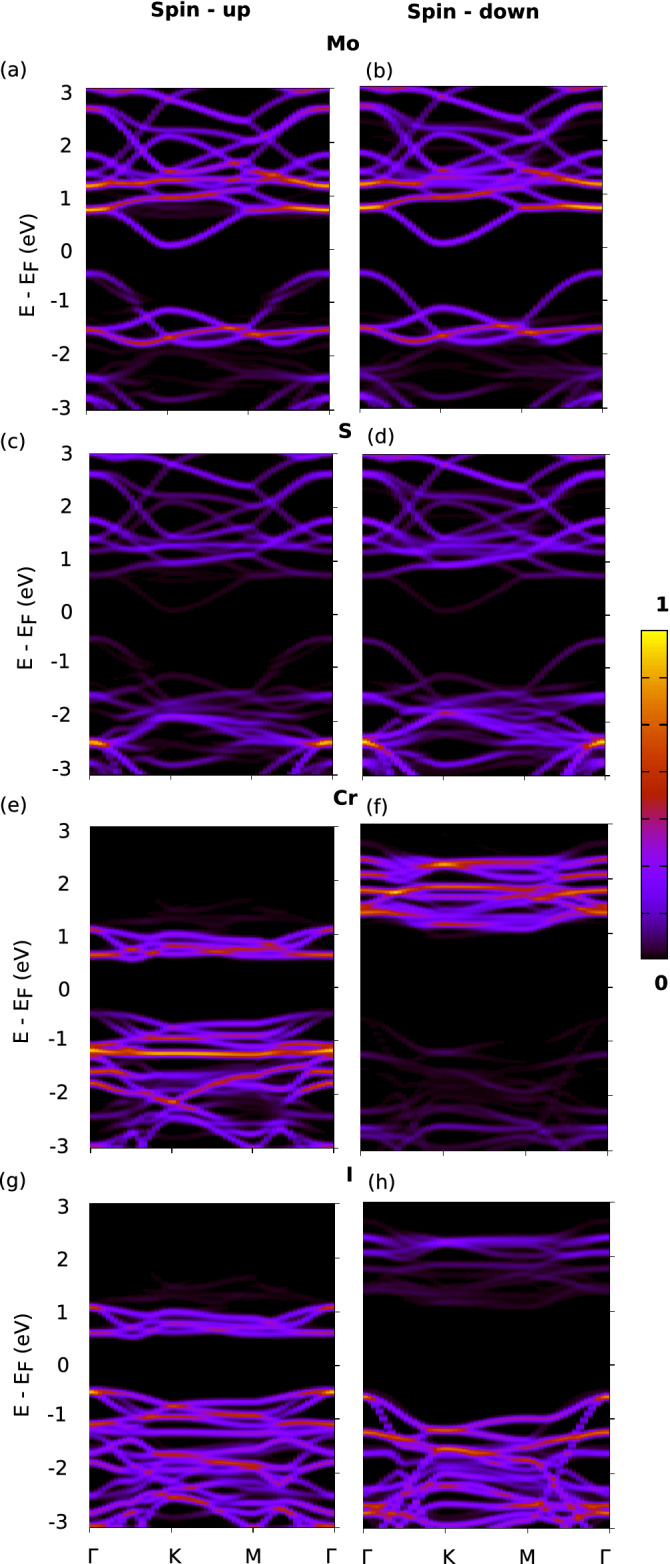
The k-resolved DOS projected on individual atom types (KDOS(k,E)) refer to “Simulation methods” section for details) is shown for (**a**,**b**) The spin up and spin down bands for 2:1 MS/CrI$_{3}$ projected on Mo states respectively. (**c**,**d**) The spin up and spin down bands projected on S respectively. (**e**,**f**) The spin up and spin down bands projected on Cr respectively. (**g**,**h**) The spin up and spin down bands projected on I respectively. The color scale has been normalized with respect to the maximum value of the state localized around their respective atom type.

In Fig. 3, we show the k-resolved DOS projected onto the atomic states and this is particularly useful when the overlap and mixing of the states are high. In Fig. 3a,c,e,g we have plotted the spin resolved bands projected on Mo, S, Cr and I for 2:1 MS/CrI$_{3}$ for the spin-up states respectively. The corresponding band projections on the spin-down states are shown in Fig. 3b,d,f,h respectively. The color scale has been normalized with respect to the maximum weight of an atomic state summed across the k-points of the Brillouin zone. The bands projected on atoms for the heterostructure system ideally shows the contribution of atom states (spin-up and down states) towards the total spin-polarized band structure shown in Fig. 2.

Figure 3a–d shows the KDOS projected on the spin up and spin down states of Mo and S respectively. One observes that Mo primarily contributes to the states above the Fermi level which weakly hybridize with CrI$_3$ below. It is also clear that MoS$_2$ remains non-magnetic upon adsoprtion. The feature regarding the reduced indirect bandgap of strained MoS$_2$ is mainly due to the shift of Mo states closer to the Fermi level. This complements the results found and highlighted in the DOS projected on atoms (Fig. 2c). In Fig. 3e–h, the KDOS projected on the spin-up and spin-down states of Cr and I are shown respectively. The spin-up states just above the Fermi level (0–1 eV) show strongly hybridized Cr and I contributions and these states weakly hybridize with the Mo states of strained MoS$_2$. The spin-down states of MoS$_2$ typically lie in the spin-down bandgap of CrI$_3$ and remain non-interacting. This reconfirms that the strain of MoS$_2$ lattice to accommodate the symmetry of CrI$_3$ below and the weak interaction of the heterostructure results in the decreased spin-independent indirect bandgap for the resulting heterostructure.

The primary conclusion from examining the electronic structure of 2:1 MS/CrI$_{3}$ would be the presence of a spin independent bandgap obtained for a ferromagnetic 2-D semiconductor. This, to our knowledge, has not been reported previously^69,70^. A phenomenological application of this system is realized in spin-resolved magnetic storage devices^71^, where the operating voltage across the two spin states would remain the same. This effect is further complemented by two aspects of this heterostructure: First is the reduced indirect band gap of 0.53 eV^72^ and second is its 2-D geometry which would allow higher density of spacial packing.

### Electronic structure of 3:1 graphene/CrI$_{3}$

In this section we discuss the spin dependent electronic and magnetic properties of $3\times 3$ graphene adsorbed on $1\times 1$ monolayer of CrI$_3$. The stable configuration of this system is when the top-left graphene ring is on top of a Cr atom below and the subsequent electronic properties are studied for this configuration (3:1 G/CrI$_{3}$).

**Figure 4:**
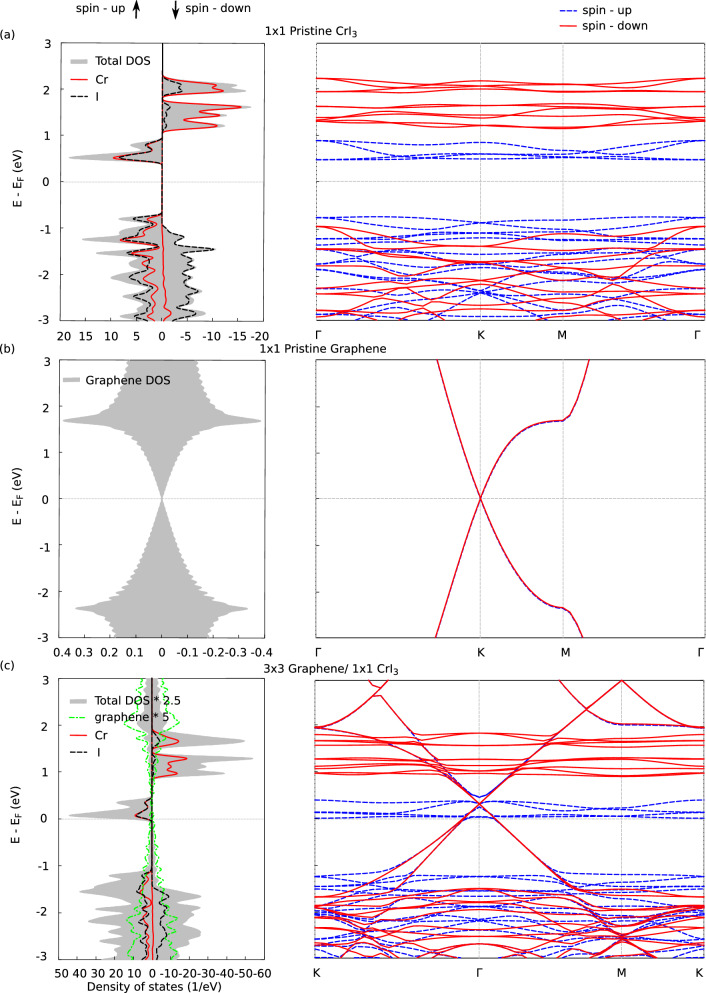
Spin-polarized projected density of states (DOS) and bandstructure for (**a**) pristine monolayer CrI$_{3}$ with $1\times 1$ unit cell (**b**) pristine monolayer graphene with $1\times 1$ unit cell (**c**) 3:1 G/CrI$_{3}$ (top configuration) with $3\times 3$ graphene adsorbed on $1\times 1$ CrI$_{3}$. Total DOS (grey) is magnified 2.5 times and DOS of C(dashed green) is magnified 5 times and Gaussian smearing of 0.004 eV has been used to visualize the DOS.

Figure 4a shows the spin-dependent DOS projected on individual atom types and bandstructure of pristine monolayer CrI$_{3}$. This has been discussed in the previous (“Electronic structure of 2:1 MS/CrI_3_” section) and included here for analytical completion of 3:1 G/CrI$_{3}$ system. In Fig. 4b, we present the spin-dependent bandstructure and DOS of monolayer graphene^73^. It displays a non-magnetic behaviour^74^, exhibits no bandgap, and has a Dirac cone at *K* point of the Brillouin zone sampled across $\Gamma$-K-M-$\Gamma$^75^. When the periodicity of $1\times 1$ graphene is extended to a $3\times 3$ supercell within an ab initio platform using a plane wave basis set^76^, we find that the Dirac cone ($\pi$ and $\pi ^{*}$) bands exactly crosses at $\Gamma$ point due to an empirical 3N rule^77^.

In Fig. 4c, we show the spin-polarized DOS and bandstructure of graphene adsorbed on CrI$_{3}$ (3:1 G/CrI$_{3}$). The resulting heterostructure is found to be metallic and magnetic with the magnetic moments primarily localized on CrI$_{3}$^52^. The atomic magnetic moment of Cr is 3.07 $\upmu _{\mathrm{B}}$/atom, I is 0.07575 $\upmu _{\mathrm{B}}$/atom and graphene only becomes slightly magnetic with atomic magnetic moment as 0.0002 $\upmu _{\mathrm{B}}$/atom. The spin-up states of CrI$_{3}$ shift closer to the Fermi level and the Dirac cone of graphene shifts above the Fermi level. The detailed spin-resolved bands calculated with the GGA+U approximations for CrI$_{3}$, 2:1 MS/CrI$_{3}$ and 3:1 G/CrI$_{3}$ are plotted in Fig. 2 of SI^57^. This can be explained by Löwdin population analysis^68^ which quantify the interfacial charge transfer. From the Löwdin population analysis we find that graphene loses an atomic charge of 0.24 electrons and CrI$_{3}$ gains an atomic charge of 0.24 electrons which is consistent with the shift in the electronic states as seen in Fig. 4c.

The I states are fully occupied and the Cr 3d$^{3+}$ states indicate why the heterostructure system is magnetic. But the $p_{z}$ orbitals of graphene remain unoccupied. To compensate electrons for the unoccupied $p_{z}$ orbitals of C atoms, all the spin-up and down states of Cr and I tend to move closer to the Fermi energy level where the deficient $p_{z}$ orbitals of C atoms exist. We can understand this further by comparing the C atomic states of pristine monolayer graphene and graphene states of 3:1 G/CrI$_{3}$. We find that the Dirac cone for the heterostructure system shifted away from the Fermi energy level by 0.075 eV. Similarly, we compared the Cr and I states of pristine CrI$_{3}$ with its counterpart of the heterostructure system. We found that the states of Cr (3:1 G/CrI$_{3}$) shift towards the Fermi energy level by 0.45 eV for the states above the Fermi level. The Cr states below the Fermi level shift away from the Fermi energy level by 0.35 eV. In case of I atoms, the highest unoccupied energy levels shift towards the Fermi energy level by 0.45 eV and for the lowest occupied energy levels they shift away from the Fermi energy level by 0.26 eV. The *d* orbitals of Cr and *p* orbitals of I interact strongly with the $p_{z}$ orbitals of C atoms of graphene thereby modifying the Dirac cone of graphene only for the spin-up states for the heterostructure system. For the spin-down case, the Dirac cone falls in the band gap of the system and remains non-interacting. This suggests that there are no quantum anomalous hall (QAH) states^78^ present for this system in its pristine form.

**Figure 5:**
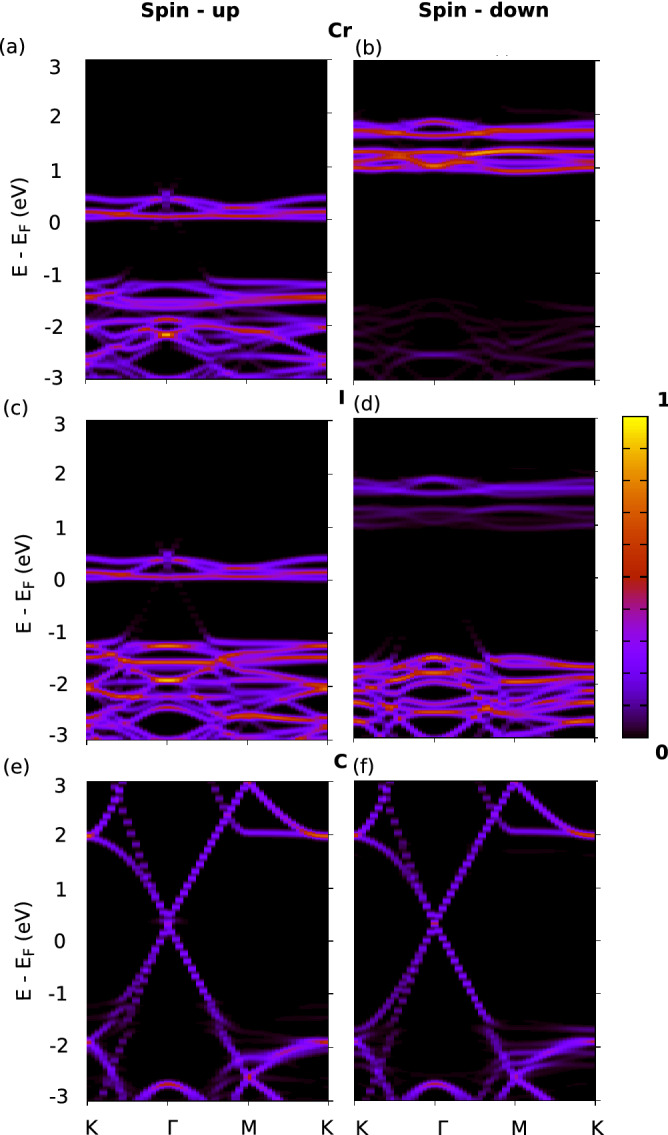
The k-resolved DOS projected on individual atom types (KDOS(k,E)) is shown for (**a**,**b**) The spin up and spin down bands for 3:1 G/CrI$_{3}$ projected on Cr states respectively. (**c**,**d**) The spin up and spin down bands projected on I respectively. (**e**,**f**) The spin up and spin down bands projected on graphene respectively. The color scale has been normalized with respect to the maximum value of the state localized around their respective atom type.

In Fig. 5, we show the spin resolved bands projected in the atomic orbitals centered around the Löwdin functions for 3:1 G/CrI$_{3}$. The spin up and spin down bands of the system projected on the atomic orbitals of Cr have been shown in Fig. 5a,b respectively. The projections of the spin up and spin down bands on I are shown in Fig. 5c,d respectively and the same on graphene are shown in Fig. 5e,f respectively. Comparing Fig. 5a,c, it is clear that the spin up states close to the Fermi level is strongly hybridized with similar contributions from both Cr and I. The spin down states for both Cr and I lie well above the Fermi level and one would expect the interaction of graphene, close to the Fermi level, is with the spin up states of Cr and I. Although graphene remains relatively non-magnetic (pointing to a weak van der Waals’ interaction between the interfaces), the Dirac cone of the spin up states of graphene hybridize with the states of CrI$_3$. The spin-down Dirac cone is in the spin-down bandgap of CrI$_3$ and does not show any hybridization. Thus we can conclude that graphene adsorbed CrI$_3$ behaves as a ferromagnetic metal with graphene showing nascent Chern insulating properties. The two primary reasons for this is the interfacial charge transfer and the hybridization of graphene with the spin-up states of CrI$_3$.

**Figure 6:**
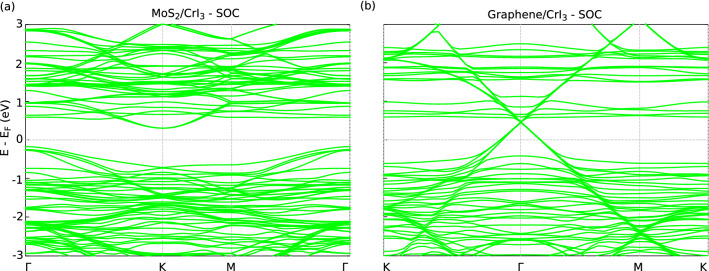
Spin-orbit coupled (SOC) bandstructure of (**a**) 2:1 MS/CrI$_{3}$ (**b**) 3:1 G/CrI$_{3}$ heterostructures.

Figure 6a shows the influence of Rashba spin-orbit coupling on the electronic properties of 2:1 MS/CrI$_{3}$ and Fig. 6b shows the same for G/CrI$_{3}$ heterostructure. It is observed in Fig. 6a that although the system remains an indirect bandgap semiconductor, the bandgap reduces to 0.47 eV. MoS$_2$ is found to show a large energy level splitting in the presence of SOC due to matrix element effects^79–81^ in the order of 170 ± 2 meV corresponding to the valence band at *K*^82^. Since we consider a strained MoS$_{2}$ weakly interacting with the substrate, the presence of SOC induces a band split of 0.01 eV corresponding to the valence band at the *K* point. The reduction in the bandsplitting is due to the strained MoS$_{2}$ and substrate effects^83^. The conduction band energy level split corresponding to the *K* point is found to be 2 meV which is in agreement with other theoretical studies^83^. Comparing the spin-polarized bands of Fig. 3a,b with SOC bandstructure Fig. 6a, we find the valence bands shift closer by 0.29 eV towards the Fermi energy level and the conduction bands shift away by 0.23 eV from the Fermi energy level.

SOC plays a pivotal role in splitting energy levels of the 3:1 G/CrI$_{3}$ heterostructure system as shown in Fig. 6b. The unoccupied conduction states of CrI$_3$ shift to higher energies by 0.57 eV and graphene decouples from the substrate. The occupied SOC induced valence states of CrI$_{3}$ shift to higher energies by a value 0.63 eV. Also these states split by an energy difference of 0.08 eV at the *K* point. The Dirac cone splits by $\sim$ 30 meV suggesting the possibility of SOC induced Chern insulating states^84^. Also the Dirac cone shifts up by 0.13 eV towards higher energy levels of unoccupied states.

We can therefore infer that the electronic structure of 3:1 G/CrI$_{3}$ undergoes a transition to a ferromagnetic metal upon adsorption. We find that in the presence of spin-orbit coupling, the Dirac cone falls in the bandgap of CrI$_{3}$ and the states begin to split. This indicates the existence of Chern insulator states which can be exaggerated by dopants or external dynamic effects^52^. This would play an important role in the design of spintronic devices with robust control over the spin-polarized electronic currents^85^.

## Conclusion

We have studied the spin-dependent electronic and magnetic properties of graphene and MoS$_2$ adsorbed on monolayer CrI$_3$. We find that when MoS$_2$ is adsorbed on CrI$_3$, the resulting heterostructure behaves like a ferromagnetic semiconductor with a significantly reduced spin-independent, indirect bandgap of 0.53 eV. The reason for the reduced bandgap ($\sim$ 70%) can be attributed to the presence of strained crystal symmetry of mono-layer MoS$_{2}$ when grown on CrI$_3$ and the weak interfacial van der Waals interactions. We attribute the reduction in the bandgap ($\sim$ 70%) to the presence of strained crystal symmetry of mono-layer MoS$_{2}$ when grown on CrI$_3$ and the weak interfacial van der Waals interactions. Graphene adsorbed on CrI$_3$ on the other hand was found to behave as a ferromagnetic metal and displays a significant interfacial charge transfer resulting in the shift of the graphene Dirac cone above the Fermi level. Introducing SOC for MoS$_2$/CrI$_3$ results in further decrease of the indirect bandgap of the system from 0.53 to 0.47 eV. The band splitting of the conduction band Mo states (strained) was found to be 2 meV. Introducing SOC to graphene/CrI$_3$ heterostructure results in the emergence of Chern insulating states with the Dirac cone splitting by $\sim$ 30 meV. The decoupling of the Dirac cone and the shift of the unoccupied CrI$_3$ states is due to the interfacial charge transfer. These heterostructures can be used to design novel spintronic devices with efficient control over the spin channels.

## Supplementary Information

Supplementary Information 1.
