# Supplementary material for: Substrate induced electronic phase transitions of CrI3 based van der Waals heterostructures
Source: Sci Rep. 2021 Jan 8;11:198. doi: 10.1038/s41598-020-80290-5 (PMC7794430; doi:10.1038/s41598-020-80290-5)
Supplement: Supplementary file 1 — Supplementary Information 1. [file 41598_2020_80290_MOESM1_ESM.pdf]

Supplementary Information for

Substrate induced electronic phase transitions of CrI_3 based van der Waals heterostructures

Shamik Chakraborty[1,*] and Abhilash Ravikumar[1]

[1]Nanoelectronics Research laboratory, Department of Electronics and Communication Engineering, Amrita School of Engineering, Amrita Vishwa Vidyapeetham, Bengaluru, India
[*]c_shamik@blr.amrita.edu

Adsorption energies and interlayer distances for the various configurations

Configuration	Adsorption energy (eV)	Interlayer Distance (Å)
**2:1 MS/CrI_3**		
Top	-0.517	3.51
Bridge	-0.464	3.60
Hollow	-0.517	3.52
**3:1 G/CrI_3**		
Top	-0.677	3.49
Bridge	-0.649	3.54
Top-Y	-0.646	3.52

Table 1. Adsorption energies and interlayer distances for different configurations of the two proposed 2-D heterostructure systems - 2:1 MS/CrI_3 and 3:1 G/CrI_3

References

1. Kokalj, A. XCrySDen—a new program for displaying crystalline structures and electron densities. *J. Mol. Graph. Model.* **17**, 176–179, DOI: 10.1016/S1093-3263(99)00028-5 (1999).
2. Webster, L., Liang, L. & Yan, J.-A. Distinct spin–lattice and spin–phonon interactions in monolayer magnetic CrI3. *Phys. Chem. Chem. Phys.* **20**, 23546–23555, DOI: 10.1039/C8CP03599G (2018).
3. Zhang, J. *et al.* Strong magnetization and Chern insulators in compressed graphene/CrI_3 van der Waals heterostructures. *Phys. Rev. B* **97**, 085401, DOI: 10.1103/PhysRevB.97.085401 (2018).
4. Kuklin, A., Visotin, M., Baek, W. & Avramov, P. CrI3 magnetic nanotubes: A comparative DFT and DFT+U study, and strain effect. *Phys. E* **123**, 114205, DOI: 10.1016/j.physe.2020.114205 (2020).

Comparision of the bandgap values using GGA, GGA+U and HSE06

Sl.No.	System	GGA (eV)		GGA+U (eV)		HSE06 (eV)
		**Up**	**Down**	**Up**	**Down**	
(i)	CrI_3	1.24 (1.132[2])	2.11	1.02 (1.19[3], ∼ 1[4])	3.3 (2.8[4])	2.12 (1.93[3])
(ii)	2:1 MS/CrI_3	0.53	0.55	0.53	0.55	-

Table 2. Energy bandgap values (E_g) for systems : (i) Pristine monolayer CrI_3 (ii) 2x2 MoS_2 adsorbed on 1x1 CrI_3 (2:1 MS/CrI_3) upon implementation of methods (a) GGA (b) GGA+U (c) HSE06

Supercell configurations of 2:1 MS/CrI_3 and 3:1 G/CrI_3

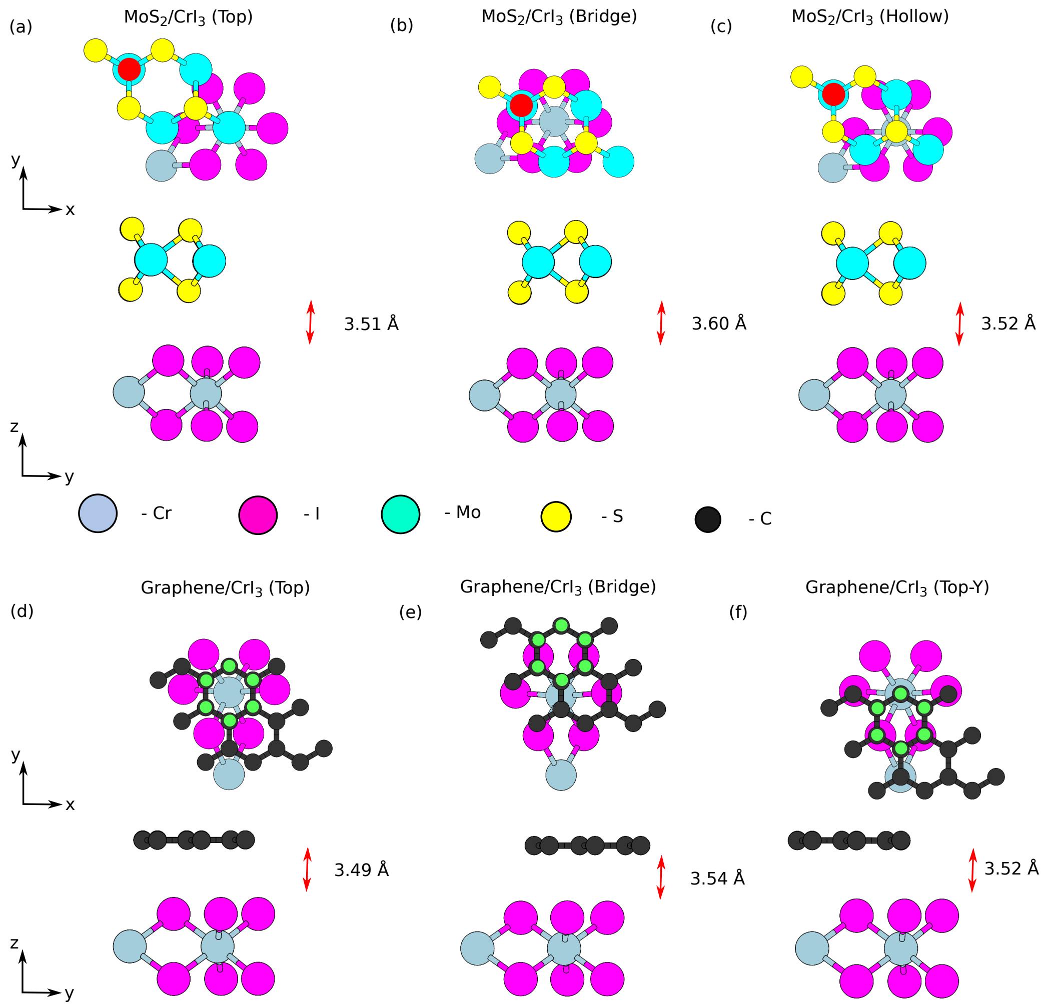

Figure 1. (a,b,c) The top and perspective view of the adsorption configurations (Top,Bridge,Hollow) of 2:1 MoS_2 on CrI_3 respectively. (d,e,f) represent the adsorption configurations (Top,Bridge,Top-Y) for 3:1 graphene on CrI_3. The configurations are visualized using XCrySDen.[1]

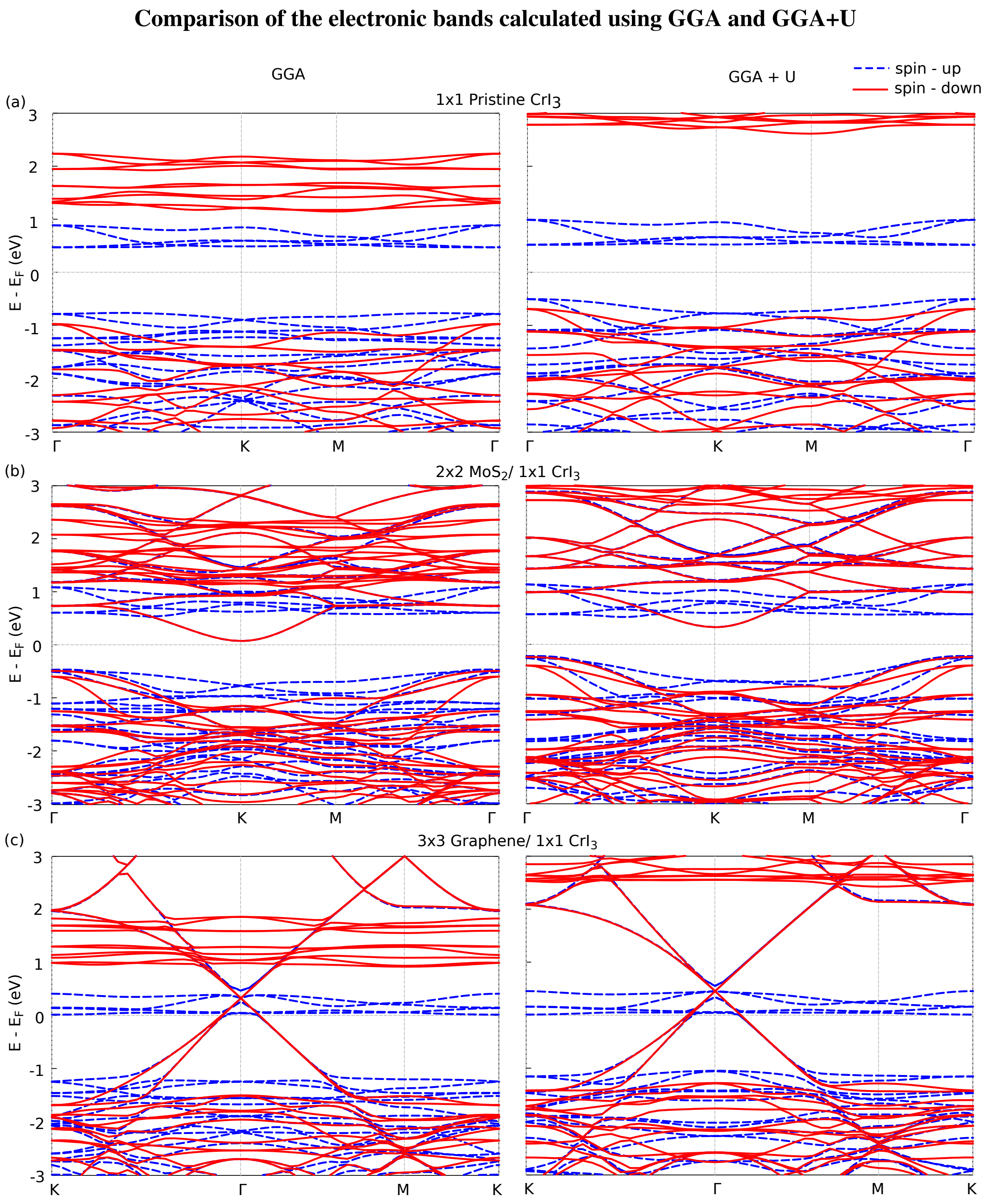

Figure 2. Comparison of spin resolved bands for GGA (left panel) and GGA+U (right panel) methods respectively (a) monolayer CrI_3 (b) 2x2 MoS_2 adsorbed on 1x1 CrI_3 (2:1 MS/CrI_3) (c) 3x3 graphene adsorbed on 1x1 CrI_3 (3:1 G/CrI_3)

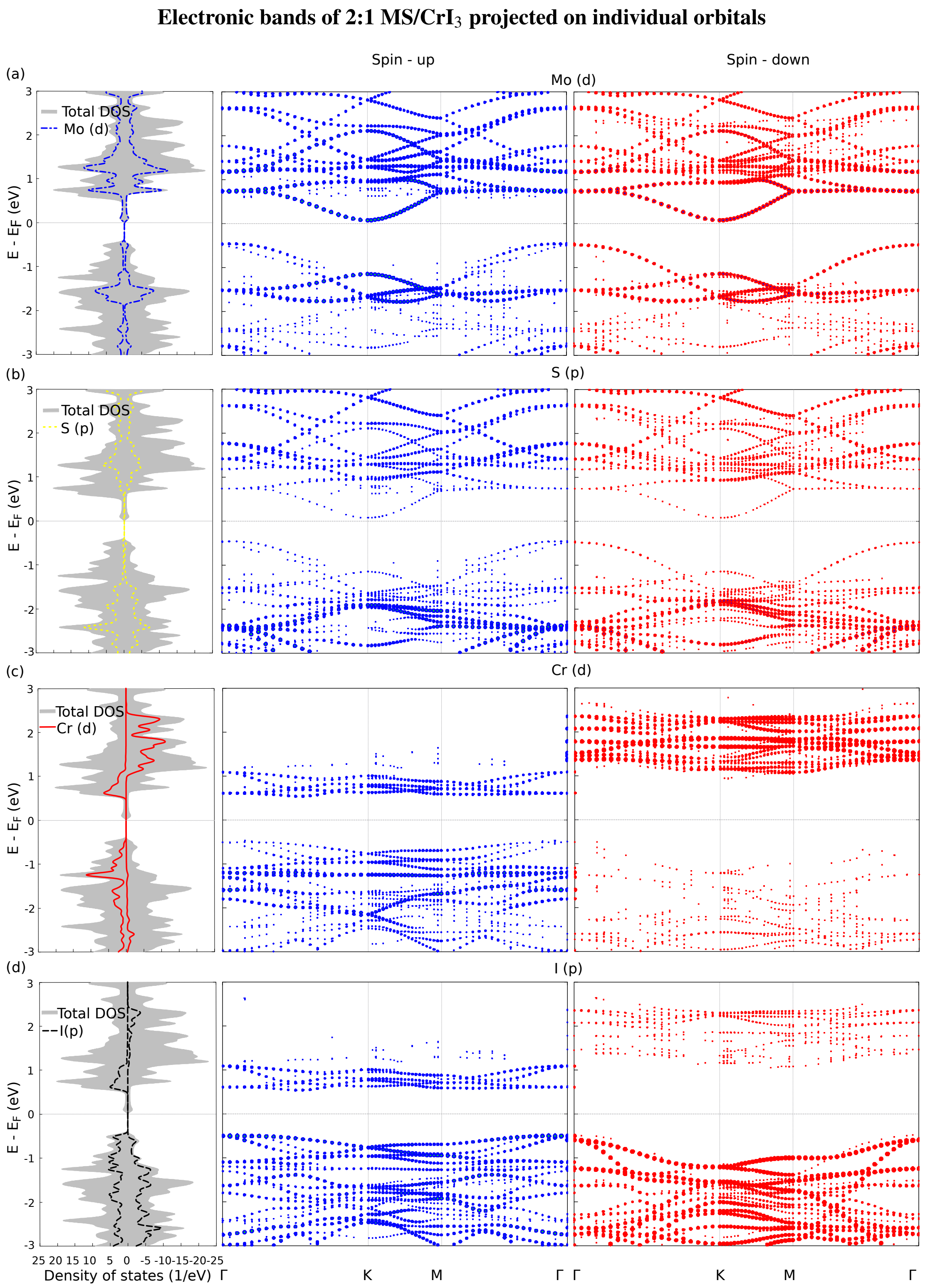

Figure 3. The spin resolved DOS and bands projected on individual orbitals for 2:1 MS/CrI_3. Spin up bands (blue), Spin down bands (red) (a) *d* orbitals of Mo (b) *p* orbitals of S (c) *d* orbitals of Cr (d) *p* orbitals of I

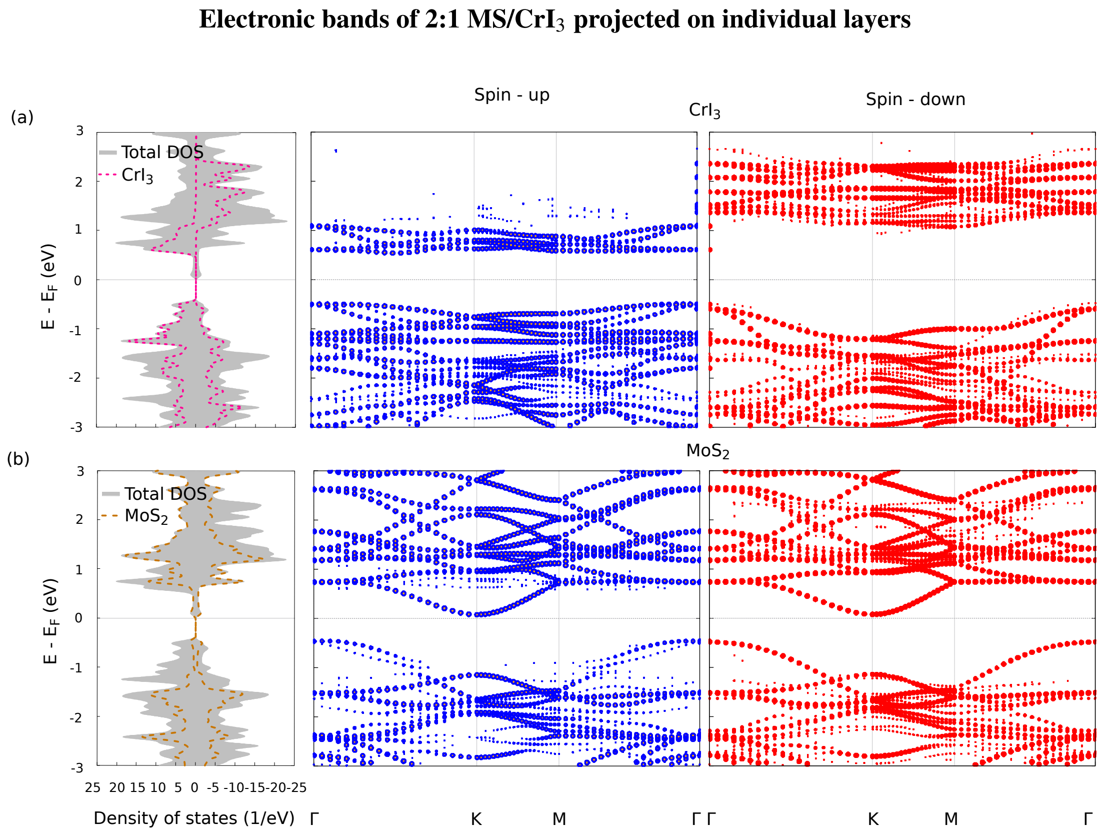

Figure 4. The spin resolved DOS and bands projected on separate heterostructure components for 2:1 MS/CrI_3. Spin up bands (blue), Spin down bands (red) (a) CrI_3 (b) MoS_2

Electronic bands of 3:1 G/CrI_3 projected on individual orbitals

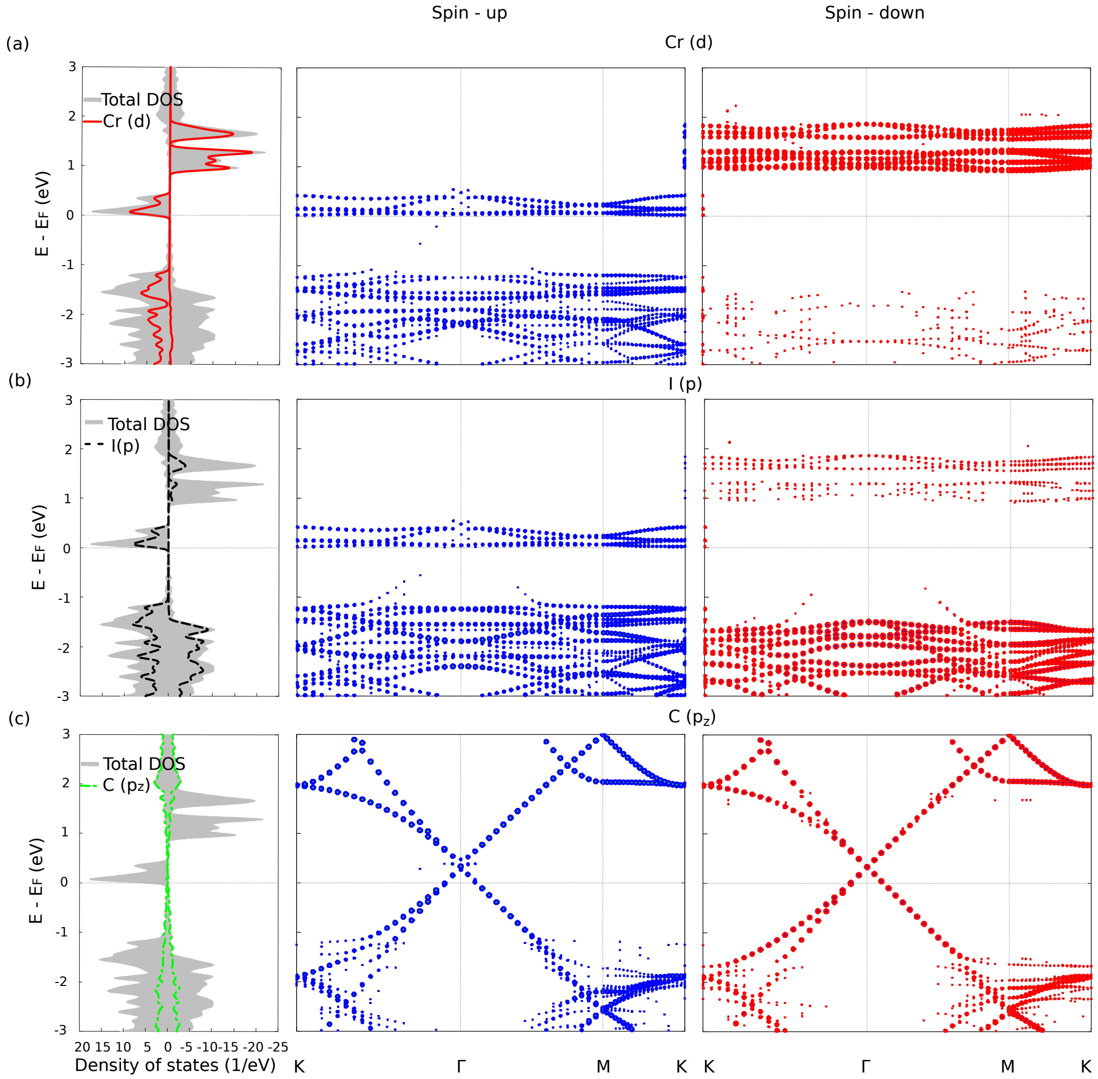

Figure 5. The spin resolved DOS and bands projected on individual orbitals for 3:1 G/CrI_3. Spin up bands (blue), Spin down bands (red) (a) *d* orbitals of Cr (b) *p* orbitals of I (c) p_z orbitals of C

Electronic bands of 3:1 G/CrI_3 projected on individual layers

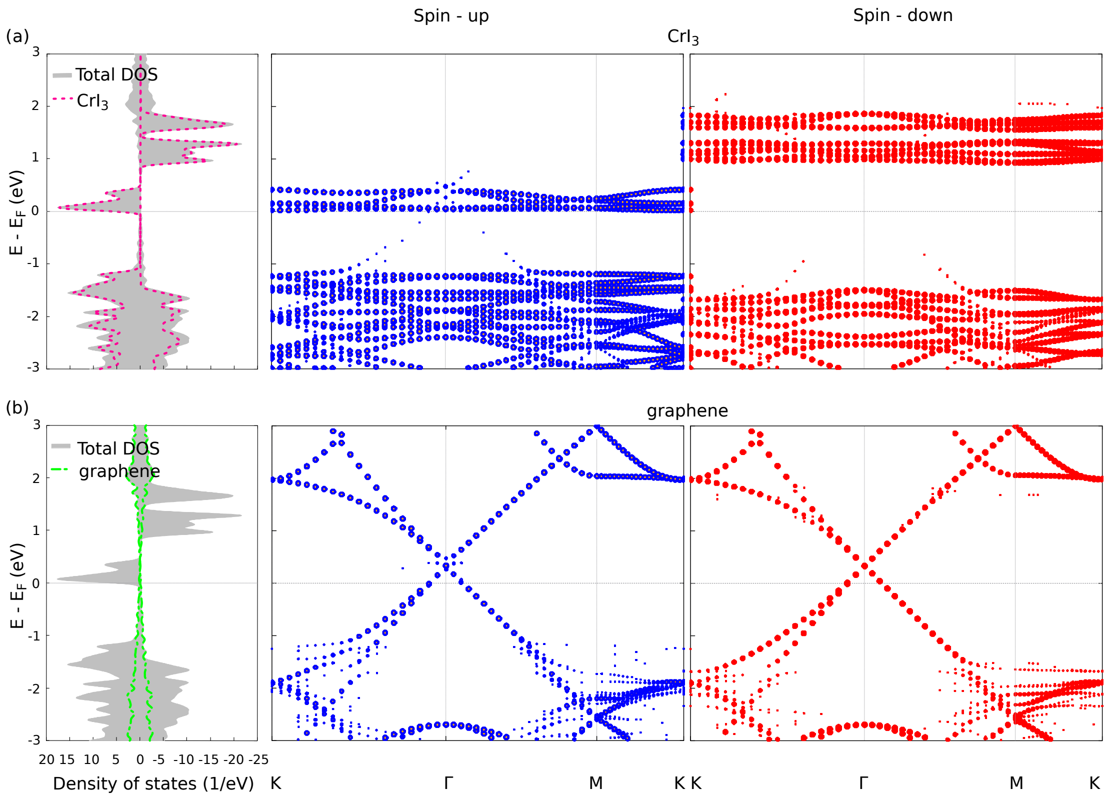

Figure 6. The spin resolved DOS and bands projected on separate heterostructure components for 3:1 G/CrI_3. Spin up bands (blue), Spin down bands (red) (a) CrI_3 (b) graphene:(C)
